# Subjective ratings of masker disturbance during the perception of native and non-native speech

**DOI:** 10.3389/fpsyg.2015.01065

**Published:** 2015-08-11

**Authors:** Lisa Kilman, Adriana A. Zekveld, Mathias Hällgren, Jerker Rönnberg

**Affiliations:** ^1^Department of Behavioral Sciences and Learning, Linköping University, Linköping, Sweden; ^2^Linnaeus Centre HEAD, The Swedish Institute for Disability Research, Linköping University and Örebro University, Linköping, Sweden,; ^3^ENT/Audiology and EMGO+ Institute for Health and Care Research, VU University Medical Center, Amsterdam, Netherlands; ^4^Department of Otorhinolaryngology, Section of Audiology, Linköping University Hospital, Linköping, Sweden

**Keywords:** perceived disturbance, native, non-native, speech maskers, noise maskers, working memory

## Abstract

The aim of the present study was to address how 43 normal-hearing (NH) and hearing-impaired (HI) listeners subjectively experienced the disturbance generated by four masker conditions (i.e., stationary noise, fluctuating noise, Swedish two-talker babble and English two-talker babble) while listening to speech in two target languages, i.e., Swedish (native) or English (non-native). The participants were asked to evaluate their noise-disturbance experience on a continuous scale from 0 to 10 immediately after having performed each listening condition. The data demonstrated a three-way interaction effect between target language, masker condition, and group (HI versus NH). The HI listeners experienced the Swedish-babble masker as significantly more disturbing for the native target language (Swedish) than for the non-native language (English). Additionally, this masker was significantly more disturbing than each of the other masker types during the perception of Swedish target speech. The NH listeners, on the other hand, indicated that the Swedish speech-masker was more disturbing than the stationary and the fluctuating noise-maskers for the perception of English target speech. The NH listeners perceived more disturbance from the speech maskers than the noise maskers. The HI listeners did not perceive the speech maskers as generally more disturbing than the noise maskers. However, they had particular difficulty with the perception of native speech masked by native babble, a common condition in daily-life listening conditions. These results suggest that the characteristics of the different maskers applied in the current study seem to affect the perceived disturbance differently in HI and NH listeners. There was no general difference in the perceived disturbance across conditions between the HI listeners and the NH listeners.

## Introduction

Listening in noisy environments can be strenuous for one and all. Even so, people seem to differ in their subjective evaluation of the impact of disturbing sounds on speech perception. This may be due to a variety of factors and knowledge of these factors provides insight into how individuals experience listening in challenging situations. One relevant individual factor is hearing acuity, i.e., whether the individual is normal-hearing (NH) or hearing-impaired (HI). Individuals with hearing loss are more likely to have difficulties in difficult listening situations than NH individuals (McCoy et al., 2005; Tun et al., 2009; Zekveld et al., 2010, 2011). Other aspects that might affect the outcome are age and cognitive functions, as well as the characteristics of the target and the background maskers.

In this study we evaluate how NH and HI listeners perceive disturbance of different types of maskers (stationary, fluctuating, babble Swedish, and babble English) in native and non-native languages.

Previous research indicates that some types of background maskers are considered more challenging than others (Pichora-Fuller, 2009). For example, speech perception in fluctuating maskers is experienced more demanding than listening to speech in stationary maskers (Pichora-Fuller, 2009). It is also known that HI listeners have more difficulties to listen “in the dips” that exist in fluctuating maskers than NH listeners (Festen and Plomp, 1990; Versfeld and Dreschler, 2002). Human speech though, appears to have a special position as a background sound, in particular when it is intelligible. In fact, subjective ratings of perceived disturbance have been found to be associated with the intelligibility of ambient speech maskers; the higher the intelligibility, the higher the disturbance ratings (Venetjoki et al., 2006). However, in objective measures of performance, several studies have confirmed that when the background speech consists of an unfamiliar language or less well mastered language, the result is usually a release in masking (Rhebergen et al., 2005; Van Engen and Bradlow, 2007; Calandruccio et al., 2010; Van Engen, 2010; Gautreau et al., 2013; Kilman et al., 2014). Furthermore, in the study of Calandruccio et al. (2013), when the background speech consisted of linguistically and phonetically distant (English target and Mandarin masker) versus close (English target and Dutch masker) language pairs; the listener performance increased when the distance increased. In this study, we do not measure objective performance but subjective ratings and it is not for certain that performance and perceived disturbance reflect matching result.

Perceived disturbance is influenced by several factors: it is partly based on difficulties in separating similar signals (Brouwer et al., 2012) and partly on the meaningful content of the distracting speech (e.g., Pichora-Fuller, 2009). Speech in background maskers might also be perceived differently for HI listeners as compared to the listeners, due to the hearing impairment *per se*. Moore (1985) argued that impaired temporal and spectral resolution is a key factor explaining the difficulties experienced by HI individuals to understand speech in background sounds.

It has been suggested that persons with hearing-impairment have to invest more processing resources to recognize spoken words than individuals with NH (Rabbitt, 1991).It is likely that this additional investment may contribute to the fatigue experienced by HI individuals at the end of the day. Research regarding this topic shows that individuals with hearing loss need more time after work to rest and recovery (Nachtegaal et al., 2009).

When an individual is focusing on a conversation and this conversation is disturbed by competing sound, it is plausible that the attention of the individual is captured by the interfering sound (Mattys et al., 2012). Yet, it is also plausible that the individual tries to re-focus his/her attention on the conversation. However, this may require a “cost” associated with dividing attention and separating the sound and the target signals (Mattys et al., 2012). Such processing could increase the level of attentional effort, i.e., the effort it takes to ignore the distracter and selectively attend to the target (Mattys et al., 2012; Koelewijn et al., 2014).

In the current study, the aim was to assess perceived *disturbance* from a masker during speech perception. We suggest an association between perceived *disturbance* and perceived *effort*. Effort is here assumed to be a consequence of perceived disturbance. Listening *effort* has been defined as “the mental exertion required to attend to and understand an auditory message” (McGarrigle et al., 2014). Listening may become effortful as a result of background noise, hearing impairment (McGarrigle et al., 2014) and/or being a non-native speaker of the target language (Mattys et al., 2012). The definition of “*disturbance*” is “the interruption of a settled and peaceful condition” (Oxford English Dictionary). In the context of the current study (i.e., speech perception) the definition of disturbance is: “The interruption of intended listening.” As a result, the attentional focus may be directed toward the interrupting sound. It has been claimed that the degree of auditory disturbance, i.e., the ability to control attention and avoid distraction, can be attributed to individual differences in working memory capacity (Conway et al., 2001; Kane et al., 2001; Sörqvist et al., 2012). High working memory capacity individuals seem to have more steadfast focus of attention and less processing of the background sound (Sörqvist and Rönnberg, 2014).

The relationship between working memory and language understanding is explained in the framework of ease of language understanding (ELU; Rönnberg, 2003; Rönnberg et al., 2008, 2013). Generally, the model clarifies the relationship between implicit and explicit functions during language processing. Furthermore, the mismatch function in the model explains the concept of perceived disturbance. When the listening situation is relatively undisturbed, the incoming semantic signal can be matched to the stored language representations in long-term memory. In that case, lexical access proceeds implicitly with ease, and language understanding is established. However, if the language signal is degraded by noise, hearing impairment and/or a non-native language, a mismatch may occur and the listener will have difficulties understanding the message. The more degraded the signal is, the more likely that the listener will experience the mismatch as more disturbing. Or expressed differently: The degree of mismatch outlines the degree of perceived disturbance. Additionally, for degraded speech, listeners will have difficulties to find language representations in the long-term memory and will as a consequence have to employ explicit processing in an attempt to comprehend the message. Thus, working memory must be invoked in order to succeed in language understanding. The ELU model describes that the degree of listening effort is related to the amount of explicit cognitive resources required to disentangle the fuzziness between the language input and the stored language representations in the long-term memory.

Even though listening in noise and its negative consequences are well documented (e.g., Kjellberg et al., 1996; Larsby et al., 2005; Jahncke and Halin, 2012; Hua et al., 2013), the main focus in studies applying subjective noise- and disturbance-ratings is usually the impact of environmental sounds. For example, the disturbance of office noise and traffic/railway/aircraft noise is commonly assessed. Furthermore, previous studies within the field of speech perception have focused on listening effort and how it can be measured objectively (Kramer et al., 1997; Murphy et al., 2000; Tun et al., 2009; Zekveld et al., 2010) and subjectively (Larsby et al., 2005; Zekveld et al., 2010). Studies in speech perception measuring self-rated disturbance are sparse and have mainly focused on simulated workplace-settings, like office noise, daycare and traffic settings (Hua et al., 2014), or perceived effort and disturbance when completing a task in office noise (Hua et al., 2013). To our knowledge, there is currently no empirical study of subjectively rated masker disturbance during the perception of masked native and non-native speech.

In the present study, we therefore evaluated the perceived disturbance for NH and HI listeners perceiving Swedish and English target speech in different masker conditions, including stationary and fluctuating noise and two-talker babble in Swedish and English. The subjective ratings analyzed in the present study were collected in the context of a larger study (Kilman et al., 2014, 2015).

Hearing impairment is commonly associated with increased listening disturbance (Hua et al., 2014; Skagerstrand et al., 2014).Therefore, we hypothesized that HI listeners will experience the different speech and masker conditions as generally more disturbing than the NH listeners.

Speech is generally considered to be more interfering than other sound sources (Venetjoki et al., 2006). Consequently, we hypothesized that both NH listeners and HI listeners will rate the speech maskers as more disturbing than the two noise-maskers in both target languages. Interactions between target language, masker conditions and hearing status were expected, but there is no firm theoretical basis for the exact pattern of disturbance.

## Materials and Methods

### Participants

Forty-three participants; 22NH (12 females and 10 males) and 21 HI (12 females and 9 males) were recruited for the study. In the NH group, the ages ranged from 28 to 64 years (*M* = 49.5, SD = 9.8) and in the HI group, the ages ranged from 28 to 65 years (*M* = 50.1, SD = 10.2). There was no significant difference in age between the NH group and the HI group [*t* (41) = 0.25, *p* = 0.804]. The NH participants were recruited from workplaces in Linköping and the HI from the audiology clinic at Linköping University Hospital, Sweden. In the NH group, education ranged from 11 to 21 years (*M* = 15.8) and in the HI group, education ranged from 8 to 21.5 years (*M* = 13.7). There was a significant difference in education between the NH group and the HI group [*t* (40) = –2.15, *p* < 0.05].

All participants were native Swedish speakers and had learned English as NH children in the Swedish school-system. Additional inclusion criteria for the HI participants were that they had an acquired bilateral, symmetrical sensorineural hearing loss with no severe tinnitus complaints. The study was approved by the regional ethics committee in Linköping and all participants provided written informed consent. All testing took place at Linköping university hospital and the participants received a small gift for taking part in the study.

### Stimuli and Tests

#### Pure Tone Audiometry

**FIGURE 1:**
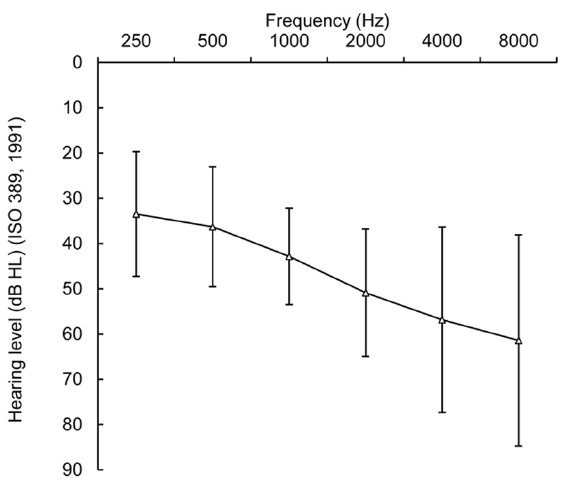
**Means and SDs (error bars) of the unaided pure-tone audiometric thresholds of the hearing-impaired participants.** Hearing thresholds are averaged over both ears.

Pure-tone average thresholds of the NH and HI participants at the frequencies 500, 1000, 2000, and 4000 Hz were measured in the beginning of the test session. The NH participants had pure tone hearing thresholds of a maximum of 20 dB HL between 250 and 2000 Hz and a maximum of 35 dB HL at 4000 Hz. One participant had a threshold of 45 dB HL at 4000 Hz in one ear. For the HI participants, the average hearing threshold across frequencies (PTA_4_) was 46.7 dB HL (SD = 10.7 dB HL). The PTA_4_ ranged from 25.0 dB HL to 71.3 dB HL. The average degree of hearing loss varied from slight (16–25 dB; *n* = 1) through mild (26–40 dB; *n* = 6), moderate (41–55 dB; *n* = 11), moderately severe (56–70 dB; *n* = 2) to severe (71–90 dB; *n* = 1) (Clark, 1981) (Figure 1).

#### SRT in Noise and Speech

The SRT test was used to measure sentence intelligibility (Plomp and Mimpen, 1979) in Swedish (Hällgren et al., 2006) and in American English HINT (Nilsson et al., 1994). The HINT sentences are short and ordinary, phonemically balanced and grouped in 25 lists with 10 sentences in each. The HINT sentences were recorded with a male native speaker in Swedish and a male native speaker in English. Eight conditions were employed; two target language conditions, Swedish and English and four masker conditions; stationary masker, fluctuating masker, two-talker babble Swedish and two-talker babble English (see description below). Every condition consisted of 20 sentences and the conditions were counterbalanced across the participants. Every sentence was used only once. The masker onset occurred 3 s before speech onset and masker off-set was 1 s after speech off-set. For the NH participants, the speech was presented at a fixed level of 65 dB SPL. For the HI participants, the presentation levels of the target speech and masker were off-line adapted according to the Cambridge prescription formula (Moore and Glasberg, 1998) based on pure tone thresholds of the best ear. A stepwise two-up-two-down adaptive procedure (Plomp and Mimpen, 1979) was to determine the level of the masker for each sentence, targeting an SNR required to perceive 50% of the sentences correctly.

*The stationary masker* was a speech-shaped noise developed by Nilsson et al. (1994) and by Hällgren et al. (2006).

*The fluctuating masker* was created from the speech-shaped noise of the target language with the same envelope fluctuations as the two-talker babble in Swedish or English (depending on the target language). The envelopes were extracted by applying a low-pass filter with cut-off frequency of 32 Hz (for details see Agus et al., 2009). Two fluctuating maskers were used, one was matched spectrally to the Swedish target and temporally to the Swedish babble and one was matched spectrally to the English target and temporally to the English babble.

*Two-talker Babble Maskers* The Swedish two-talker babble was created by mixing the soundtracks from a native female and a native male reading Swedish newspapers. The English two-talker babble was created by mixing the soundtracks from one native British English male and one American English female reading English/American newspapers.

#### Subjective Ratings

The participants were instructed to rate the perceived listening disturbance immediately after completing each condition. The participants were given a sheet of paper with eight scales, one for each condition and were asked to answer the following question: “*How disturbing was the noise you just heard?*” The question was the headline on the paper. The disturbance rating scales ranged from 0 to 10 on a continuous scale, where 10 represented “extremely disturbing” and 0 “not disturbing at all.”

## Results

The means and standard deviations of the perceived disturbance in the eight different SRT conditions are shown in Table 1. The most disturbing masker for the HI listeners seems to be the *babble Swedish in the Swedish target language*. The most disturbing masker for the NH listeners seems to be the *babble Swedish in the English target language*.

**TABLE 1:** **The means and standard deviations of the perceived disturbance in the eight different SRT conditions**.

	**Swedish target**				**English target**			
	**Stat**	**Fluc**	**BS**	**BE**	**Stat**	**Fluc**	**BS**	**BE**
HI	6.5(1.7)	6.8(1.7)	7.9(1.2)	7.0(1.6)	7.0 (1.7)	7.1 (1.5)	6.9 (1.4)	7.5 (1.5)
NH	6.0(1.8)	6.3(1.8)	6.7(1.8)	6.3(1.7)	6.2(1.8)	6.3(1.9)	7.3(1.8)	6.8(2.1)

HI, Hearing-impaired listeners; NH, Normal-hearing listeners; Stat, Stationary noise; Fluc, Fluctuating noise; BS, Babble Swedish; BE, Babble English.

Analysis of variance (ANOVA) was conducted to assess the *impact of the two target languages (Swedish and English) and the four masker types (stationary noise, fluctuating noise, babble Swedish and babble English)* as within participant factors on the perceived disturbance for HI listeners and NH listeners (i.e., the between-participant factor). The ANOVA showed a main effect of *masker type*; *F* (3,123) = 5.4, *p* < 0.05, eta squared = 0.12, suggesting a moderate to large effect, but no main effect of hearing status. Also, a *significant three-way-interaction between group, language and masker type* was observed; *F* (3,123) = 6.53, *p* < 0.001, eta squared = 0.14, suggesting a large effect. The result indicates that the interaction effect between target language and masker type differed between the NH listeners and the HI listeners, as generally expected. Follow-up analysis of simple effects showed that there was a significant interaction between *target language* and *masker type* for the HI listeners; *F* (3, 60) = 6.8, *p* < 0.001, *d* = 0.25, suggesting a small significance (For the calculation of d from dependent *t*-test, we used the formula described in Dunlap et al., 1996, s 171). There was no significant interaction for the NH listeners; *F* (3, 63) = 1.6, *p* = 0.19. This result reflects that for HI listeners, there was a difference in perceived disturbance *between the maskers for the Swedish and English target languages*. No significant effects were found of target language; *F* (1, 41) = 1.64, *p* = 0.13, or group, as between-participant factor; *F* (1, 38) = 3.7, *p* = 0.06.

We expected the *speech maskers (Swedish and English babble in both target languages)* to be perceived more disturbing than the *noise maskers (stationary and fluctuating maskers in both target languages)*. We tested whether this was the case separately for the NH listeners and HI listeners. For the NH listeners, the *speech maskers* were perceived as more disturbing than the *noise maskers*; *t* (21) = 2.57, *p* < 0.05, *d* = 0.34, suggesting a small to moderate significance. However, for the HI listeners, the *speech maskers* were not perceived as more disturbing than the *noise maskers*; *t* (20) = 1.65, *p* = 0.114.

*HI Listeners* Probing the overall three-way interaction further, a *post hoc*, pairwise comparison (Bonferroni adjusted for multiple comparison at the 0.05 level) of the differences in disturbance ratings between the masker types across languages confirmed a significant difference for the HI listeners for the *Swedish babble*, *between* the *Swedish* and the *English target languages*; *t* (20) = 4.70, *p* < 0.001, *d* = 0.81, suggesting a large significance. This demonstrates that the perceived disturbance for the HI listeners in the *Swedish babble* was larger for *Swedish* as compared to the *English target language*. None of the differences in perceived disturbance of the other masker types (i.e., *stationary noise*, *fluctuating noise*, and *babble English*) between the two target languages were statistically significant; *t* (20) = –1.20 to –1.76, all *p* > 0.05.

**FIGURE 2:**
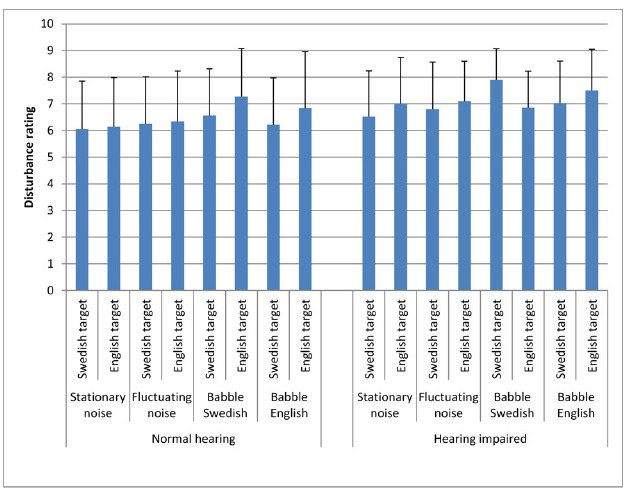
**Means and SDs of the perceived disturbance ratings for the NH and HI participants in Swedish and English target with stationary noise, fluctuating noise, babble Swedish, and babble English**.

Significant differences (Bonferroni adjusted for multiple comparison at the 0.05 level) were shown between the *Swedish babble* and each of the other maskers (*stationary*, *fluctuating*, *English babble*) for the *Swedish target language*, *t* (20) = 2.7–3.9, *p* < 0.05, *d* = 0.93 (SweBS/SweSt), *d* = 0.73 (SweBS/SweFl), *d* = 0.60 (SweBS/SweBE), suggesting a moderate to large significance for the differences. The result indicated that the HI listeners perceived *the Swedish babble* as more disturbing than the other three maskers in *Swedish target language*. No significant differences were found between the maskers for the *English target speech*; *t* (20) = 1.45–2.17, all *p* > 0.05 (Figure 2).

*NH Listeners* The same *post hoc* pair-wise comparisons were performed on the data of the NH listeners (independent *t*-tests with Bonferroni adjustment for multiple comparison at the 0.05 level). There were no significant differences in perceived disturbance from the maskers between the two *target languages* for the NH listeners. For the *English target language*, the results show significant differences between the *stationary masker* and the *Swedish babble*, *t* (21) = 3.5, *p* < 0.05, *d* = 0.62, suggesting a moderate significance, and between the *fluctuating masker* and the *Swedish babble*, *t* (21) = 3.0, *p* < 0.05, *d* = 0.50, suggesting a moderate significance. These results indicate that the perceived disturbance of the *Swedish babble* was larger than that of the *stationary* and the *fluctuating* maskers for *English target language* (Figure 2).

## Discussion

The main aim of this study was to explore how NH and HI listeners perceived disturbance in four different background conditions in their native and non-native languages, respectively. We expected the HI listeners to experience more listening disturbance than the NH listeners. This was not the case, as the current data did not show a statistically significant difference in perceived disturbance between the HI and the NH listeners, although a trend was observed (*p* = 0.06) with relatively high disturbance ratings by the HI listeners.

We also expected the speech maskers to be perceived as more disturbing than the noise maskers. The result confirmed our prediction for the NH listeners but not for the HI listeners. Although the HI listeners perceived a high level of disturbance from the Swedish babble for Swedish as target speech, the Swedish babble for English target speech was not perceived as more disturbing than the other maskers, including the noise maskers. For English as target speech, the NH listeners perceived the Swedish babble as more disturbing than both noise maskers. The characteristics of the maskers applied in the current study seem to affect the perceived disturbance differentially in HI and NH listeners.

Generally, the disturbing effects of interfering speech can be explained in terms of two mechanisms. First, linguistic similarity (Brouwer et al., 2012) between the target speech and the masker speech affect the degree of disturbance, and secondly, the intelligibility of the words in the masker speech affects masker disturbance. Additionally, the disturbing effect of interfering speech is commonly ascribed to higher cognitive processing levels than that of interfering noise. Interfering speech captures attention, induces semantical interference, and is often associated with increased cognitive load (Cooke et al., 2008; Mattys et al., 2009; Koelewijn et al., 2012).The degree of disturbance seems to depend on the lexical familiarity with the masker. Larger interference is observed if the masker has semantically noticeable meaning (c.f., cocktail party effect, Cherry, 1953). The NH listeners in the current study may have overheard more native, familiar words in the Swedish babble masker than the HI listeners. This may have temporarily captured their attention (Conway et al., 2001). For the English target speech/Swedish babble condition, it may have been cognitively more demanding for the NH listeners to focus on the non-native target speech while trying to inhibit speech in their native or most accomplished language.

Surprisingly, for the HI listeners the same condition (i.e., the English target/Swedish babble) was equally disturbing as the disturbance from the other three maskers for English target speech. For the HI listeners, the specific features of the different maskers do not result in differences in perceived disturbance for this non-native target speech: the masking effects of the four maskers are equivalent. One inference to be drawn from this is that the HI listeners most likely had difficulties to perceive any words from the speech maskers correctly. Therefore, the Swedish babble in the English target speech condition was not more disturbing than the other maskers. We also suggest that the HI listeners may have to invest all their processing resources (Rabbitt, 1991) to focus on the English target speech, trying to identify the words and solve the assigned task to listen to and repeat the sentences.

As mentioned earlier, the Swedish target/Swedish babble condition was the most disturbing for the HI listeners. The lack of hearing acuity is likely one reason for this result, as the impaired spectral and temporal resolution (Moore, 1985) causes a reduced ability to distinguish different sounds. Additionally, impaired spectral and temporal resolution increases the difficulty to distinguish the linguistically similar (Brouwer et al., 2012) target and masker speech. The relative similarity between the target and the masker depends on factors like phonological, semantic and/or syntactic content of the two streams. From the English target/Swedish babble condition, we suggest that HI listeners likely did not correctly perceive many words in the masker. Additionally, we suggest that the Swedish target/Swedish babble condition taps into the same level of phonological and syntactic processing and therefore produces a high level of perceived disturbance for the HI listeners.

Listeners often have better speech perception for relatively unfamiliar maskers as compared to familiar, or intelligible, native speech (e.g., Calandruccio et al., 2010). For the subjectively perceived disturbance ratings, the HI listeners obtained benefit in the Swedish target speech, as the unfamiliar masker (the English babble) was not perceived as more disturbing than the stationary and the fluctuating noise. In the English target speech, the English babble was not perceived as more disturbing than any of the other maskers. The NH listeners did not perceive the English babble as more disturbing than any of the other maskers in the Swedish target speech. However, in English target speech there was no difference between the speech maskers, as the NH listeners perceived both speech maskers (familiar and unfamiliar) as more disturbing than the two noise maskers.

## Conclusion

There is no difference in the perceived disturbance from noise and speech maskers during native and non-native speech perception between HI and NH listeners.

For NH listeners, the perceived disturbance from the speech maskers was larger than that from the noise maskers. For HI listeners, the perceived disturbance from speech maskers was similar to that from the noise maskers.

The characteristics of the different masker types applied in the current study seem to influence the perceived disturbance differently in HI as compared to NH listeners.

### Conflict of Interest Statement

The authors declare that the research was conducted in the absence of any commercial or financial relationships that could be construed as a potential conflict of interest.
